# A Review of Natural Joint Systems and Numerical Investigation of Bio-Inspired GFRP-to-Steel Joints

**DOI:** 10.3390/ma9070566

**Published:** 2016-07-12

**Authors:** Evangelos I. Avgoulas, Michael P. F. Sutcliffe

**Affiliations:** Department of Engineering, University of Cambridge, Trumpington Street, Cambridge CB2 1PZ, UK; eia21@cam.ac.uk

**Keywords:** natural joints, adhesive joints, biomimetics, bio-inspiration, composites

## Abstract

There are a great variety of joint types used in nature which can inspire engineering joints. In order to design such biomimetic joints, it is at first important to understand how biological joints work. A comprehensive literature review, considering natural joints from a mechanical point of view, was undertaken. This was used to develop a taxonomy based on the different methods/functions that nature successfully uses to attach dissimilar tissues. One of the key methods that nature uses to join dissimilar materials is a transitional zone of stiffness at the insertion site. This method was used to propose bio-inspired solutions with a transitional zone of stiffness at the joint site for several glass fibre reinforced plastic (GFRP) to steel adhesively bonded joint configurations. The transition zone was used to reduce the material stiffness mismatch of the joint parts. A numerical finite element model was used to identify the optimum variation in material stiffness that minimises potential failure of the joint. The best bio-inspired joints showed a 118% increase of joint strength compared to the standard joints.

## 1. Introduction

In recent years, the use of composite materials in automotive and aerospace industries has shown an upward trend due to their good stiffness-to-weight (*E*/*ρ*) and strength-to-weight (*σ*/*ρ*) ratios. However, joining is a key issue in the mechanical design of composite parts due to the structural discontinuity that joints cause. This can be a source of unreliability, a factor critical in the aerospace and automotive sectors. Adhesive bonding is a popular method for joining dissimilar materials because this can produce joints with higher structural efficiency, excellent fatigue life, a particularly small weight penalty and more uniform stress fields than alternatives such as fastening or riveting. Additionally, corrosion between the dissimilar materials is prevented [1,2,3,4,5]. One of the main difficulties with joining dissimilar materials, such as composite with metals, is related to the large difference in stiffness properties between the adherends. The stiffness mismatch leads to high stress concentrations, and thus weak joints.

Nowadays, many of the modern turboprop engines in operation are equipped with carbon fibre reinforced plastic (CFRP) propeller blades. World-leading manufacturers of integrated propeller systems use CFRP-to-steel joints to attach the CFRP blade to the steel hub. Applications range from regional airliners (e.g., Bombardier’s twin-engine Q400 Dash 8) and military airlifters (e.g., Lockheed Martin’s four-engine C-130J) to marine hovercraft (Textron Systems’ Landing Craft Air Cushion (LCAC) hovercraft). Additionally, composite structures are used in piping systems with applications in a wide range of industries (e.g., aerospace, marine, chemical), where composite materials are an attractive alternative to metals considering the extreme environments that the piping systems are exposed in for onshore or offshore applications [6]. Since joining of dissimilar materials is widely used in high performance structures, an innovative approach is needed to meet this challenge.

Biomimetics uses ideas from nature to inspire engineering solutions. In the context of joining, nature has developed a wide range of solutions for joining of many different materials and geometries, which have the potential to inspire more robust and efficient joints in engineering applications. This paper expands the conference proceedings published in [7], including and expanding the literature review in that paper and drawing on the conclusions of that review to develop a novel biomimetic solution to joining of composites to metals.

The biomimetic design approach cannot consist of just copying the designs of nature because of the significant differences between natural and engineering materials. Thus, several biomimetic design reviews exist, focusing on a variety of engineering challenges where nature has provided inspiration [8,9,10,11,12,13,14,15,16,17,18,19,20]. Vertechy and Parenti-Castelli [21] proposed the following steps when using a bio-inspired approach for the design of man-made joints:

1. comprehend and analyse the functions and the structure of biological joints;
2. identify the features that may be transferred from biological to engineering joints;
3. devise the most favourable joint configuration that will highlight the identified features by considering the available technology and materials;
4. use the available technology, materials and the devised joint configuration to design optimised mechanisms for the specific engineering application.

To understand how to optimise biomimetic engineering joints, first it is important to understand how biological joints “work”. Thus, Section 2 of this paper contains an extensive literature survey and discussion with a special focus on natural joint systems and their classifications. This section draws on the conference publication of Avgoulas and Sutcliffe [7]. Emphasis was given to understanding natural joints from a mechanical point of view, so as to inspire engineers to find innovative methods of joining man-made structures. The idea of a transitional zone of stiffness used by natural joint designs is then taken up in Section 3 and adapted to different types of glass fibre reinforced plastic (GFRP) to steel engineering joints. A numerical analysis methodology is developed to improve the predicted joint strength. The paper finishes with some brief conclusions.

## 2. Natural Joint Systems

This section contains a review of natural joints. Both effectively rigid joints and joints which provide relative motion between parts are considered. The findings from the review are discussed in Section 2.2, particular as they relate to biomimetic applications in engineering, and a comparison between natural and engineering joints is made in Section 2.3.

### 2.1. Literature Review of Natural Joints

Joining methods found in nature have been classified into five different groups depending on the different joining method used: Network structures, transitional zones of stiffness, bridging connections, hooks and adhesive joints. Examples for each of these types of joint are described below.

#### 2.1.1. Network Structures

Network structures use complex geometric arrangements in order to join materials. Two examples are described here: root structures where the soil and plant components have very different mechanical properties and tree joints where the elements being attached have the same material properties.

##### Root Networks

Roots are the first structure that develops in a growing plant. Root networks provide an ingenious anchorage and stability system [22,23], and provide for storage of nutrients and water. However, their structural complexity is often underestimated as they lack visibility and they are difficult to sample [24,25]. There are two main types of root system, taproot and fibrous [26]. The taproot system is characterised by having one long, thick main root that penetrates deeply into the soil (e.g., radishes, dandelions, turnips, carrots, and cacti), which makes it hard to uproot. From the main root (the taproot), smaller and thinner roots emerge. The fibrous root system is characterised by having several similarly-sized main roots that branch numerous times and form a tangled mass of roots and soil (e.g., corn, grass and most trees). Fibrous roots do not penetrate as deeply into the soil as taproots.

##### Tree Branches

**Figure 1:**
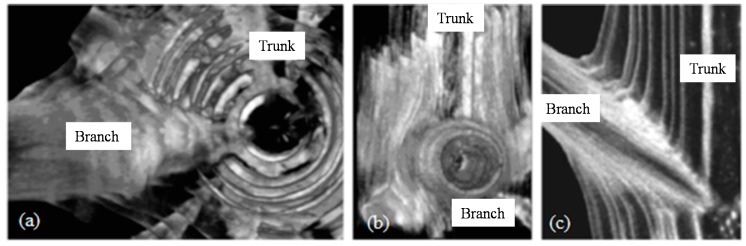
(**a**) Internal cone-shaped branch structure embedded in the tree centre; (**b**) 3D fibre lay-up; and (**c**) fibre density variation across the joint [27].

The idea of a continuous fibre flow pattern from one part of the joint to the other can be found in abundance in tree joints. The non-articulated joints that exist in trees have to withstand a combination of static and dynamic loads, including self-weight, snow and wind loading. Burns et al. [27] tested branch-trunk connections under bending from the tree species *pinus radiata*. They concluded that, despite the brittleness of the cellulose constituent of the natural wood composite, the failure mode of the tree joints was ductile. From an X-ray tomography study conducted on the branch-trunk joint [27], three key design features were identified that contribute to the mechanical properties of branch-trunk joints. These are embedded design, three dimensional (3D) fibre lay-up and variable density, as illustrated in Figure 1.

**Figure 2:**
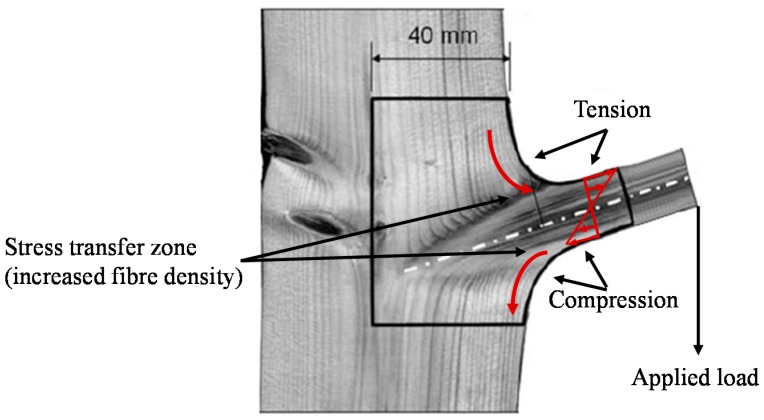
Forces and stress distribution acting on a branch-trunk joint (modified from [28]).

The embedded design consists of a cone-shaped branch end that is embedded and enclosed in the main trunk. This type of joint has the advantage of increasing the effective joint load transfer area and thus reducing the stress acting on the joint interface. Moreover, any interfacial cracks are forced to grow in mode II (shear) rather than in mode I (tension) and thus the toughness is increased. The 3D fibre lay-up is made from trunk fibres that extend forward and laterally to the joint and thus they fully enclose the branch, leading to a “ball and socket” joint configuration. Finally, fibre density variation leads to the creation of an iso-strain condition in the joint by reducing the elastic modulus mismatch between the fibres aligned with the branch and trunk directions. Figure 2 (modified from [28]) illustrates a simple example of the forces and stress distribution acting on a branch-trunk joint. Müller et al. [28] used the 3D Electronic Speckle Pattern Interferometry (ESPI) technique to directly measure strains in a mechanically loaded branch–trunk joint. The authors concluded that the branch–trunk joint in a Norway spruce tree is characterised by a homogeneous distribution of strain (iso-strain condition) achieved by a combination of naturally optimised shape, material properties and fibre orientation. This example demonstrates how nature achieves high structural efficiency using its hierarchical design, a typical finding in biological structures. Here, the nano-, micro-, meso- and macro-length scales synergistically interact in order to achieve the axiom of uniform strain [29].

Burns et al. [27] used their study of tree joints to inspire a biomimetic composite T-joint, following the embedded design concept. The biomimetic joints that they made had increased ductility and damage tolerance. However, the joints sustained lower damage initiation loads because the embedded design caused higher interlaminar stresses in the radius bend. Burns et al. [30] showed that, by optimising the stacking sequence of the plies of the composite laminate in the radius bend, the interlaminar tension and shear stresses can be reduced. This improved the onset of the damage initiation load as well as the strength and the failure displacement of the composite T-joint.

#### 2.1.2. Transitional Zones of Stiffness

The attachment of dissimilar materials is a major challenge due to the high levels of stress concentration that arise at their interface. As an alternative to geometric complexity, microstructural changes within the joint can give changes in stiffness across the interface to alleviate this stress concentration. Such transitional zones of stiffness are typified by the tendon-to-bone attachment and the mussel anchoring system described below. Another example is the hard jaws of marine polychaete worms, which anchor to soft cuticles and the ligaments at their base [31]. The jaws exhibit distinct mechanical gradients, contain molecular transition and proteins with histidine-rich domains [32].

##### Tendon-to-Bone Attachment

The tensile modulus of tendon in the direction of the muscle force is 200 MPa while it buckles in compression. By contrast, bone has a tensile modulus of 20 GPa in both tension and compression [33]. To avoid stress concentrations associated with this large change in stiffness, the tendon-to-bone insertion site is a functionally graded material with regard to its mechanical properties, extracellular matrix composition (EMC), structural organization, and mineral content [34,35]. The tendon-to-bone insertion site is additionally secured by the complex interdigitation of the calcified fibrocartilage layer with the adjacent bone [36].

**Figure 3:**
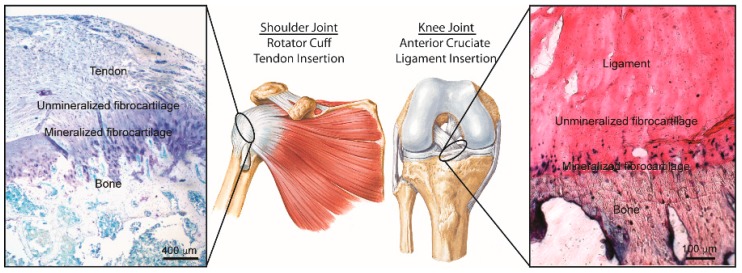
Morphology of the supraspinatus tendon-to-bone insertion site [38].

Four discrete types of tissue can be recognized in the tendon-to-bone insertion site under an optical microscope [37]. These are tendon, fibrocartilage, mineralized fibrocartilage, and bone (Figure 3) [38]. The first zone consists of tendon proper, with properties similar to those found at the mid-substance of the tendon. It consists of well aligned collagen type I fibres with small amounts of the proteoglycan decorin [39]. The beginning of the transition from tendinous to bony material is marked by the second zone, which consists of fibrocartilage. It consists of collagen types II and III, with small amounts of collagen types I, IX, and X, and small amounts of the proteoglycans aggrecan and decorin [40,41,42]. The third zone consists of mineralized fibrocartilage that indicates the transition towards bony tissue. In this zone, collagen type II is predominant. Collagen type X and aggrecan also exist in significant amounts [40,41,42]. The fourth zone consists of bone. It is mainly made up of collagen type I with a relatively high mineral content.

Two major factors contribute to the tissue stiffness increase from the tendon to bone material. Firstly, the linear increase in mineral concentration in collagen fibres stiffens the partially mineralized collagen fibres [35,43]. Secondly, a reduction of the orientation distribution of the collagen fibres in the transition from tendon to bone leads to tissue stiffening. The combination of these two factors leads to stiffness variation across the insertion site, providing a way that nature attaches two dissimilar materials through a functionally graded material composition. This gradual transition region eliminates stress singularities, provides a smooth stress distribution, and thus reduces the stress concentrations, improving the bond strength and decreasing the risk of fracture [44].

##### Mussel Anchoring System

One of the main defence mechanisms of mussels is their anchoring system, which is achieved by byssus threads [45]. Byssus threads are produced in a byssus gland located in the base of the mussel’s foot. While the thread is still liquid the tip of the foot presses onto the substratum and forms an adhesive disc. The adhesive disc attaches the thread to the substrate. To provide greater adhesion, the shape of the disc of a *mytilus edulis* mussel thread depends on the nature of the substratum [46]. The adhesive disc can be flat and expansive on coarse and rock sediments, or have a more three-dimensional form on fine sediments. Stress failures such as tearing within the pad, loss of adhesion or peeling can occur at the attachment disc [47].

The byssus thread consists of two mechanically district regions; the proximal region, which is highly extensible and wrinkled, and the less extensible and smooth distal region [48]. Each region contains a different collagen type, with typical collagen amino-acid compositions [49]. The ends of the chains of the stiffer distal part of the thread are comprised of silk-like domains. On the other hand, the ends of the chains of the less stiff proximal part are comprised of elastin domains. In the cells between the distal and proximal parts, there is a gradation of collagens with silk or elastin blocks. Biomechanical and scanning electron microscopy (SEM) studies [50] have shown that the byssus thread is a mechanically graded fibre with significant difference in stiffness throughout its length. The structural differentiation between the elastic proximal end and the stronger and stiffer distal end provides wave and water movement energy absorption and strong anchoring, even in the most wave-exposed coastal areas.

#### 2.1.3. Bridging Connections

While the above examples have been concerned with more-or-less rigid joints, there are many situations in anatomy where relative movement between components is needed, particularly but not always associated with locomotion. These joints typically use bridging connections, for example ligaments, to provide the joining mechanism. But as well as movable joints, there are immovable joints which also use bridging connections to form the joint. The following examples from the human anatomy illustrate the range of solutions that nature provides in these situations. There are three primary types of joints in the human body, fibrous (immoveable or partially moveable), cartilaginous (partially moveable) and synovial (freely moveable) [51].

##### Fixed Joints

Fibrous joints have collagen fibres that span the space between the parts. A gomphosis is an immovable fibrous joint attaching tooth roots to the alveolar sockets in the jaw bones. The fibrous periodontal ligaments bridging the gap consist of connective tissue of collagen fibres [52].

Sutures are another form of immovable fibrous joint that connect two stiff skeletal components to each other via a thin layer of a dense fibrous connective tissue (compliant interfacial seam), and thus provide flexibility to accommodate growth, respiration and/or locomotion [51,53,54,55,56,57]. Suture joints have been investigated both experimentally [53,54] and numerically [55,58], showing the correlations between the mechanical properties and the degree of interdigitation, commonly measured with the suture complexity index (SCI) [54,59]. Understanding the underlying mechanisms influencing the mechanical behaviour of suture joints is of significant interest for a wide variety of fields [56] such as mechanics [60,61,62,63], materials [64], biophysics [65], mechanical design [66], evolutionary biology [67] and biomimetics [68].

The nail is an opalescent window through to the vascular nail bed [69]. Anchoring of nails is achieved by nail matrix (nail root), which can be divided into three main parts [70,71]; the dorsal matrix, the intermediate matrix and the ventral matrix (nail bed). The strongest site of attachment of the nail plate to the nail apparatus is the nail bed [72]. The vertical arrangement of the collagen fibres of the nail bed creates a ligament-like connection between the epidermal basement membrane and the phalangeal periosteum [73,74].

##### Partially Moveable Joints

A syndesmosis is a partially movable type of fibrous joint connecting two bones together by an interosseus membrane (ligament that holds two bones together) [51]. Syndesmoses can be found between the radius and ulna and between the tibia and fibula. The joint allows the tibia and fibula to work together as a unit in the lower leg, while still permitting some motion of the joint.

Cartilaginous joints are partly moveable joints, where the bones are connected to each other by pads of either fibro cartilage or hyaline cartilage [51]. There are two main types of cartilaginous joints; synchondroses and sympheses, which are temporary and permanent joints, respectively. Synchondroses joints exist at the ends of long bones. The articulating surfaces of the bones are bound by hyaline cartilage. In long bones, the diaphysis and the epiphysis are separated by the cartilaginous plate. Examples of sympheses are the joint between pubic bones [75,76] or the attachment between vertebrae in the vertebral column by a band of fibrocartilage ring. These joints are characterised by being able to maintain stability, because only minimal motion can occur. The vertebral column is able to extend and flex due to the combination of these small movements.

##### Highly Moveable

Synovial joints are highly movable joints and they are the most common classification of joints within the human body [77]. They all have a synovial capsule (collagenous structure) surrounding the entire joint, a synovial membrane (the inner layer of the capsule) which secretes synovial fluid (a lubricating liquid) and cartilage known as hyaline cartilage which pads the ends of the bones. Synovial joints can be classified into three categories, depending on the degree of freedom of movement that they permit (i.e., uniaxial, biaxial and triaxial). Depending on the joint shape, they are further classified into six types. These are hinge [78,79,80,81,82], pivot, ball and socket [83,84,85,86], saddle [87,88,89], ellipsoidal [90] and gliding [91,92].

#### 2.1.4. Hooks

The most well-known biomimetic example inspired by nature itself is the invention of Velcro fasteners [93]. It was invented by George de Mestral in 1948 who was inspired by how a burr, with its series of tiny hooks, stuck so tenaciously to his dog’s fur. It can be seen under the microscope that the joint is based on the attachment between the tiny burr’s hooks caught by the hair of the animal’s fur. In nature, the hook-like structures in plants serve two main functions [94]. These are to support stems in a densely occupied environment [95] and to interlock with animal fur and feathers for fruit and seed dispersal [96]. The structure of the burrs’ hooks and the interlocking attachment and separation forces that they produce when they attach to the fur of animals has been studied by various researchers [96,97,98,99].

#### 2.1.5. Adhesives

In legged insects/animals the main way to obtain reaction forces, and thus locomotion, on various substrates is by the attachment organs. This plays a more significant role when animals move on steep, vertical or even inverted substrates, where adhesive forces are needed to prevent them from falling down [100]. Gecko Tape [101] was inspired by the ability of gecko lizards [102,103,104] and some spiders (e.g., *evarcha arcuate* [105]) to adhere to surfaces (independently of the orientation of the surface) because of their millions of microscopic hairs existing on their toes. Van der Waals forces are exerted from these flexible, tiny hairs and as a result they provide a powerful adhesive effect [106,107].

These attachment organs can be classified into claws, hairy pads and soft smooth pads [108,109]. Claws have been studied both experimentally and mathematically, focusing on the relationship between the attachment forces of claws, their geometry, and substrate roughness [110]. Claws may attach reliably only when the mean radii of the protrusions of the substrate are larger than the diameter of the claw tips. By contrast, such attachment is unreliable on smooth substrates [110,111,112,113]. Thus, to attach reliably on various inclined rough surfaces, many insects have evolved both claws and adhesive pads on their feet [100]. Adhesive attachment organs such as hairy pads were developed by flies [114], beetles [115] and geckos to generate adhesion by van der Waals or capillary forces [116], while tree frogs [117,118,119], ants [120,121,122,123] and crickets [124] use soft smooth pads to generate adhesive forces through capillary interactions. Song et al. [100] found that the synergy effect between the claws and adhesive pads leads to much stronger attachment forces, as compared to the action of claw or adhesive pads or even to the sum of both.

#### 2.1.6. Insect Wing Joints

Insect wings have to withstand a combination of bending and torsional deformations during flight. Wing deformability is of significant importance for the flight performance of insects and can define their flight capabilities [125,126]. Wootton [127] and Newman [128] were among the first researchers who described the influence of the wing design on the aerodynamic performance of insects. These authors showed how members of the Odonata order (i.e., dragonflies and damselflies) use both active and passive mechanisms with complex structures to control their deformations in flight. Passive mechanisms play a most important role in the flight capabilities because of the wing architecture [128] and its material properties/composition [129,130]. According to Rajabi et al. [129], the morphological adaptations that are currently known and allow passive control of wings deformations are the venation pattern [131], venational fractures [127], vein joints [128], thickened areas [127], fold and flexion lines [132,133,134], material gradients [135,136,137], and spikes located in the vicinity of joints [128,135].

According to Fauziyah et al. [138,139], dragonfly wings are structurally stable due to their venous framework, which form joints at vein-to-vein cross-over points. These cross joints can contribute to arrest cracks that might occur at the thin membranous films, typically along the longitudinal axis of the wing [140]. The veins are layered composite structures [141] mainly made from chitin (stiff) and resilin (soft rubber-like protein) [142]. Resilin is highly elastic [143] and dominates in mobile joints. Thus the joint has the ability to return undamaged to its original state after flight-induced deformation [137]. The main purpose of resilin is to absorb and store mechanical energy, providing elasticity to the system [144]. From species-to-species, venous patterns are varied and from joint-to joint-the resilin is distributed in different geometrical arrangements and volumes and across the span of the wing [138].

### 2.2. Discussion of Natural Joints

The literature survey above has described a range of different natural joints, divided into different joining methods. A theme common to a wide range of systems is how nature uses structural and material complexity to ensure effective anchorage and stress dissipation in the joint site. Parallels between different natural joints can be found related to this theme.

#### 2.2.1. Structural Complexity

There are many examples where nature uses complex architecture, taking advantage of the way that it can build in complexity from the bottom-up generation of structures. Such complexity can serve to increase the structural efficiency of the joint and reduce the material investment, since this comes with a biological cost. The majority of the material serves other functions apart from the load-carrying requirement [145]. Thus, in both plant and tendon systems, a relatively small proportion of the plant/tendon participates in the anchorage system. The corresponding imperative in engineering systems is to increase joint efficiency so as to minimise the extra weight, or perhaps cost, associated with the joint.

The root and tree network structures typify an obvious structural complexity, with the root structure serving both a joining function and providing a mechanism to draw water and nutrients from the soil. Looking at the gecko lizard, it relies on a complex hierarchical structure for the adhesive properties of its feet. Structural complexity is similarly found in the tendon-to-bone attachment with bony spicules radiating in all directions to facilitate load transfer. Even on a larger scale, tendons and ligaments take advantage of structural complexity by not forming an isolated attachment onto a bone, but instead blending several overlapping attachment sites to produce more stable anchorage [146]. In Achilles and patellar tendon attachments there is a substantial anisotropy of superficial trabeculae that can be likened to a taproot of a tree. And, despite the fact that the trabecular network is usually disregarded when tendon-to-bone attachment is considered, its structural geometry plays an essential role in tendon-ligament anchorage and stress dissipation [34].

An additional strategy is to introduce geometric complexity in the form of scalloping at the interface to increase the bonding between tissues. This approach is used for both the dentino-enamel junction and the tendon-to-bone attachment [147].

#### 2.2.2. Material Complexity

A closely related way of increasing joint efficiency in nature is to use microstructural or material complexity, again using the sophisticated generation routes available for natural materials.

The tree trunk branch offers a botanical example of such an approach, using variations in fibre density to give an iso-strain condition in the joint. Moreover, the transition zone solutions used in tendon-to-bone and mussel attachments take this material complexity further by using significant changes in local material composition and microarchitecture to ensure a smooth load transition and shock-loading capability. Similarly the dentino-enamel junction uses a mineral content gradient to increase the interfacial strength [147].

#### 2.2.3. Taxonomy Chart of Natural Joints

**Figure 4:**
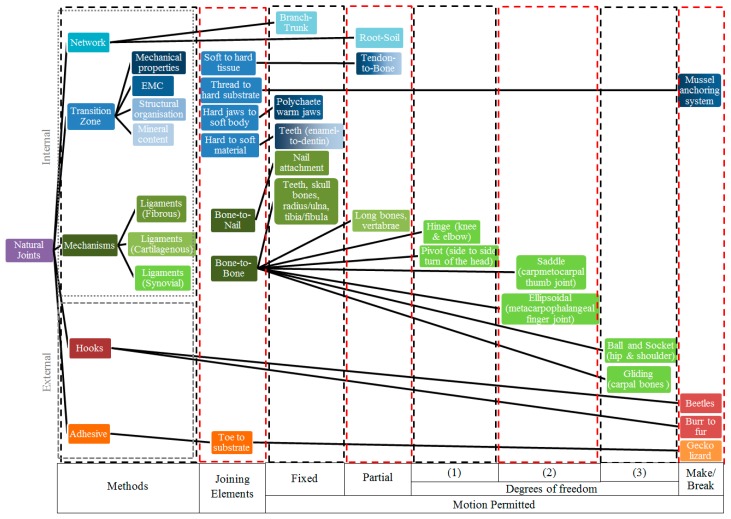
Taxonomy of natural joints.

Figure 4 shows a taxonomy chart covering the range of joints discussed above. The aim of this chart is to categorise joints in nature between dissimilar materials taking account of the different methods/functions used. The columns in the chart separate the joining methods, the joining elements of each method, and the motion permitted in each joint. The methods column defines an association of each of the five joining methods listed with corresponding colours. Shades associated with the subdivisions of the transition zone and miscellaneous joining methods are given in the right-hand side of the methods column. Across each row individual examples of natural joint systems are linked with different joining elements and permitted motions. The colours of these examples correspond to the colours defined by the methods column. Because some of the joints use more than one joining method, the joint box can be equally divided into parts with different colours. For example the mussel anchoring system uses mechanical properties and EMC in a transition zone, and so is coloured in the mechanical properties and EMC shades of blue. Similarly, the tendon-to-bone box consists of all the four different blue shades, as it uses all these transition zone methods. The chart illustrates how there are solution methods common to a range of situations, but also that there is a diversity of methods with the details adapted to the individual case.

### 2.3. Comparison between Natural and Engineering Joints

Both natural and man-made structures require joining of materials with severe property mismatches. There are two options in engineering to avoid interfacial failure in these circumstances. Firstly, the interaction energy can be increased across the interface. This is achieved by surface treatment (primers and coupling) of the metal adherends prior to bonding [148] or through bio-inspired surface texturing [149,150,151,152]. Secondly, sharp boundaries between the dissimilar materials can be avoided by manufacturing functional gradients [44]. This is a new method that creates materials with graded mechanical properties that are able to resist damage more effectively than their homogeneous counterparts. Using gradients in man-made structures has several advantages, such as reducing stress concentrations by eliminating stress singularities and developing smooth stress distributions, so as to increase fracture toughness and improve bonding strength. Nature has evolved similar strategies to join tissues with dissimilar bulk material properties. Adopting the first manufacturing strategy of increasing the interaction energy, the byssus thread of mussels contain specially modified amino acids that form charge-transfer chelate complexes with surface oxides of the rocks and metals that they are attached to [153,154]. The second manufacturing strategy of functional grading, exactly parallels the tendon-to-bone attachment, where the two zones of fibrocartilage between tendon/ligament and bone contribute to the stress dissipation at the attachment site by ensuring a gradual change in the mechanical properties from a soft tendon or ligament to a hard bone tissue, as detailed in Section 2.1.2 [155].

In summary, a biomimetic solution using a transitional zone of stiffness offers the potential to provide a robust and efficient joint for engineering situations where there is a large material stiffness mismatch. The challenge is to take these ideas from nature and to adapt them to practical engineering joints. This is what the next section aims to do.

## 3. Bio-Inspired GFRP to Steel Joints

The idea of a transitional zone of stiffness between dissimilar materials used by natural joint designs is applied in this section to adhesively bonded single-lap, double-lap and hybrid joints. The aim is to evaluate the hypothesis that using the bio-inspired design strategy of a transitional zone of stiffness across the overlap length of GFRP to steel joints can increase the strength of the joints.

The present study was triggered by the positive results obtained in previous work by the authors, where a numerical investigation of carbon fibre reinforced plastic (CFRP) to steel single lap joints (SLJ), using materials with linear elastic properties in the numerical models, was carried out [156]. The proposed SLJs considered a transitional zone of stiffness in the joint site to reduce the material stiffness mismatch. All the proposed biomimetic solutions reduced the asymmetry of the stress distribution along the bondline. By increasing the stiffness reduction in the metal part of the joint, the stress reduction at the end of the bondline was increased with a maximum shear stress reduction of 59%.

One way to achieve the stiffness variation in the overlap region of engineering joints is to gradually decrease the stiffness of the metal part of the joint by perforating it. The concept of reducing the stiffness of a steel plate using perforations was firstly proposed and patented by Unden and Ridder [157]. Related studies have been conducted by Melogranaa and Grenestedt [158], where perforated stainless steel to glass fibre reinforced vinyl ester composite joints with different surface preparations, adhesives and primers were experimentally investigated. Cao and Grenestedt [159] experimentally tested a co-infused sandwich structure with composite (glass fibre) skins joined to a perforated steel hybrid structure. The perforated concept was applied to co-infused perforated steel to CFRP hybrid joints by Avgoulas and Sutcliffe [160]. The hybrid joints were numerically and experimentally investigated under static mechanical testing. Compared to non-perforated joints, the CFRP-to-perforated joints showed a 175% increase of joint strength [160]. Similar studies that remove material from the adherend with the larger stiffness to increase the joint strength have been carried out by Hart-Smith [161], who was the pioneer of the development of adhesively-bonded scarf and stepped-lap joints with dissimilar adherends. Sato and Ikegami [162] analytically and experimentally investigated the strength of single-lap and scarf joints between CFRP and steel adherends. They found that for equal adherend thickness and a lap length to adherend thickness ratio (*l*/*t*) less than 5, the scarf joints showed a 65% to 150% increase of joint strength compared to the single-lap joints. However, when *l*/*t* was around 10, the failure strength of the single-lap joints exceeded that of scarf joints.

**Figure 5:**
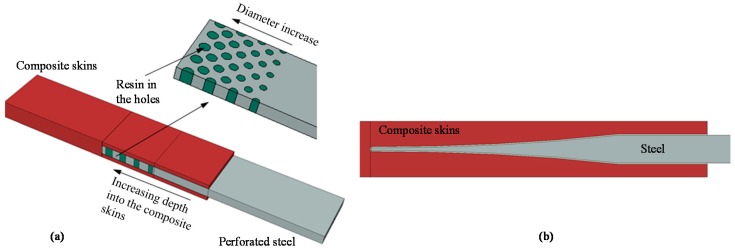
Example of: (**a**) a composite to perforated steel joint; and (**b**) composite to non-linear taper steel scarf joint.

The novelty of the present study is that a numerical finite element model is used to identify the optimum variation in material stiffness which maximises the strength of the joint. The assumption is that such an approach can be realised in practice using a steel perforation pattern (Figure 5a) or a non-linear steel taper (non-linear scarf joint; Figure 5b) that follows the optimum material stiffness variation, leading to an engineering joint design with an increased strength. 

**Figure 6:**
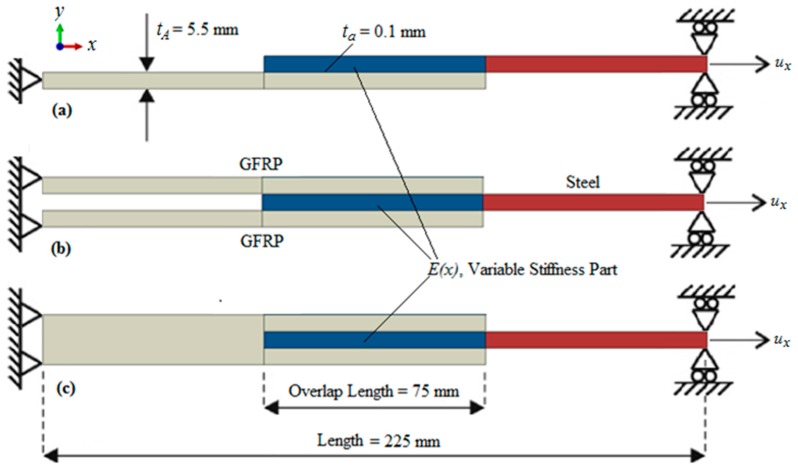
Geometry, coordinate system, materials and boundary conditions: (**a**) single-lap; (**b**) double-lap; and (**c**) hybrid joint.

Figure 6 illustrates the three joint configurations considered for the present study: single lap joints (SLJ), double lap joints (DLJ) and hybrid joints. The composite part of the joints was modelled as a unidirectional GFRP material with a Young modulus in the loading direction *E_1GFRP_* = 20 GPa. The Young modulus of the steel material was taken as 200 GPa and the Poisson ratio as 0.28. The proposed solutions offered a transitional zone of stiffness in the overlap region (OL) of the metal part to reduce the material stiffness mismatch at the joint site. Different sets of variable stiffness functions were investigated to optimise the material stiffness variation, identifying the stiffness function which minimises potential failure of the joint. The stiffness *E*(*x*) of the metal part was varied as a function of the position *x* along the OL of the joint, from a minimum value of *E*_Smin_ at the left end, to a maximum value of *E*_Smax_ at the right end of the joint. The value of *E*_Smax_ was held fixed at 200 GPa, while *E*_Smin_ was chosen to take values of 10, 20, 40, 70 or 100 GPa. The variation within the overlap between these extreme stiffnesses at the two ends was described using parabolic and s-shaped functions, with parameters *a* and *b* defining their shape as described in detail in the Materials and Methods Section below. It is believed that, in practice, non-linear scarf joints can be more reliable than the perforated steel configuration when the minimum stiffness of the steel (*E*_Smin_) has to reach very low values (*E*_Smin_/*E*_Smax_ less than 10%).

### 3.1. Materials and Methods

#### 3.1.1. Finite Element Model Implementation

The composite part of the joints was modelled as a unidirectional GFRP material with material properties given in Table 1. To calculate accurately the load carrying capacity of the adhesive joints, cohesive elements were used for the simulation [163,164]. The adhesive was modelled with a single layer of four-node, two-dimensional cohesive elements (COH2D4) of thickness 0.1 mm, compatible with the CPE4I elements used for the GFRP and steel parts [165]. A triangular cohesive zone degradation formulation was chosen because of its simplicity, widespread use for investigation purposes, especially for brittle adhesives [166], and availability in Abaqus. The material properties used to define the cohesive law are summarised in Table 2 [166,167], using the properties for the adhesive Araldite AV138 (Huntsman Advanced Materials Ltd, Cambridge, UK). A quadratic nominal stress criterion was used to define damage initiation and an energy power-law (quadratic) mixed-mode criterion was used to define damage evolution. A geometrically non-linear static general analysis was performed in Abaqus/Standard (Dassault Systèmes, Paris, France). Fixed boundary conditions were applied to the GFRP end of the joint. A displacement *u*_x_ was applied to the steel end of the model together with a lateral restraint.

#### 3.1.2. Stiffness Variation in Overlap

**Figure 7:**
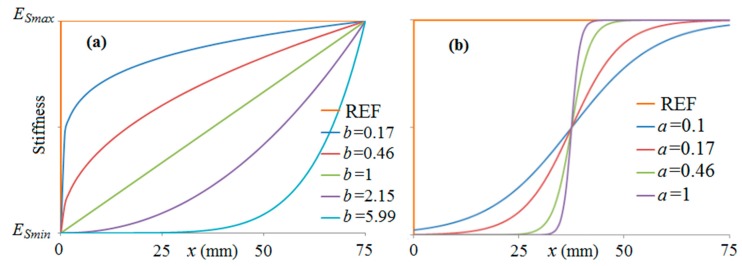
Functions used to define the variable stiffness of the steel within the overlap length for the chosen values of *a* and *b*. The steel stiffness varies from *E_Smin_* (which is a variable parameter) to *E_Smax_* (which is always equal to 200 GPa): (**a**) parabolic; and (**b**) *s*-shaped functions.

The stiffness variation as a function of position *x* within the overlap region between the extreme stiffnesses at the two ends was described using parabolic and *s*-shaped functions as defined by Equations (1) and (2), respectively.

(1) $$E(x)\;=\;E_{Smin}+(E_{Smax}-\;E_{Smin})\;{\left(\frac{x}{x_{c}}\right)}^{b}$$

(2) $$E(x)=E_{Smin}+\frac{E_{Smax}-E_{Smin}}{1+e^{-a\;\left(x\;-\;x_{c}/2\right)}}$$

where *x_c_* is the overlap length equal to 75 mm. The locations with *x* = 0 and *x_c_* = 0.075 correspond to the left and right end of the overlap length, respectively. The parameters *a* and *b* defining the shape of the curves were spaced logarithmically. These stiffness variations were chosen for their simplicity to investigate the effect of the transitional zone of stiffness on the joint strength. Future research should include approaches such as topology optimisation to identify the best stiffness variation case. Figure 7 illustrates the resulting stiffness variations within the overlap length for the chosen values of *a* and *b*. The user subroutine “USDFLD“ in Abaqus was used to implement this stiffness variation. For comparison, reference joints were modelled with the same configurations but without any stiffness variation.

### 3.2. Results

**Figure 8:**
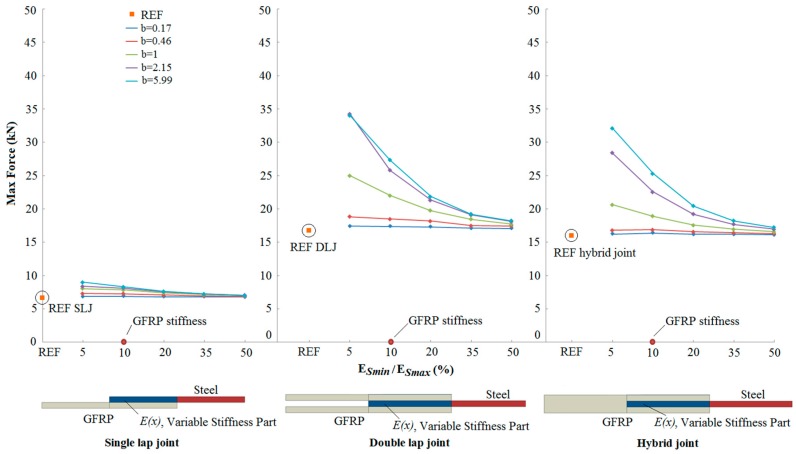
Comparison of the numerical maximum load between reference (REF) and bio-inspired joints with parabolic stiffness variations.

**Figure 9:**
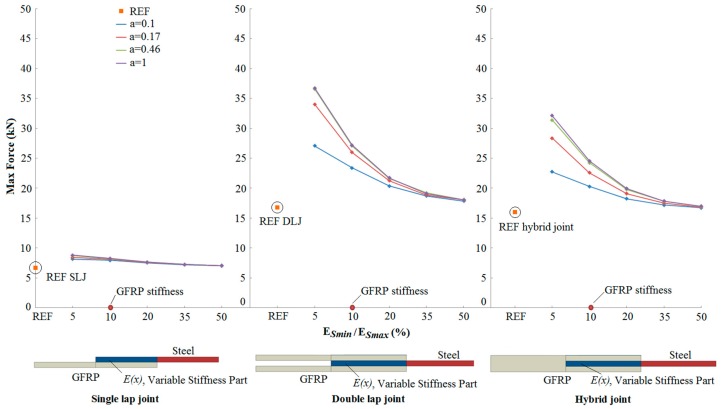
Comparison of the numerical maximum load between reference (REF) and bio-inspired joints with s-shaped stiffness variations.

Figure 8 summarises the results for the three joint configurations of the predicted failure strength, for a parabolic distribution of stiffness in the overlap region. Results for different values of the minimum stiffness *E*_Smin_ and *b* shape parameter are plotted and compared with the reference configuration without stiffness variation in the OL. The horizontal axes are separated into three parts, each section describing one of the joint configurations. The horizontal axes show the minimum stiffness (*E*_Smin_) as a percentage of the maximum stiffness (*E*_Smax_) in the steel part of the joint within the reduced-stiffness overlap length, corresponding to the different values of *E*_Smin_ considered. Thus a value of *E*_Smin_/*E*_Smax_ equal to 50% corresponds to *E*_Smin_ equal to 100 GPa, while a 10% value relates to calculations where the steel was reduced down to the stiffness of the GFRP adherend (20 GPa) at the end of the overlap region. A set of curves giving the variation of strength with stiffness reduction are given for the chosen range of values of the parameter *b* defining the shape of the variation in stiffness through the OL. For comparison with the proposed bio-inspired joints, the maximum loads of the “REF” reference joints (i.e., with no stiffness variation in the OL) are included on Figure 8. Corresponding results for the s-shaped variation in stiffness reduction are given in Figure 9 for a range of values of *a* defining the stiffness variation.

From Figure 8 and Figure 9, it can be seen that the joint strength increases as the reduced stiffness of the steel adherend approaches the stiffness of the GFRP adherend. There is a significant effect of the form of the stiffness variation on the strength, and the potential for increased strength is greater in the double lap and hybrid joints, compared with the single lap joint. Finally, for all the cases, *s*-shaped stiffness variation functions give higher (or similar) joint strengths compared to parabolic stiffness variation functions with the same stiffness range. SLJs with the stiffness in the overlap varying from 10 to 200 GPa (*E*_Smin_/*E*_Smax_ equal to 5%) and following the s-shaped function with *a* = 1 show a 32% increase of the maximum load compared to the reference joints. On the other hand, there is a 118% and 100% strength increase for DLJs and hybrid joints comparing to the reference joints, respectively, for the optimum stiffness variation. These increases in strength for the GFRP-to-steel joints are comparable with measured increases of 175% for perforated CFRP-to-steel joints [160] and between 65% and 150% for some CFRP-to-steel scarf joint configurations [162]. Similar qualitative improvements were noted by Hart-Smith [161], although no direct comparison figures were provided.

**Figure 10:**
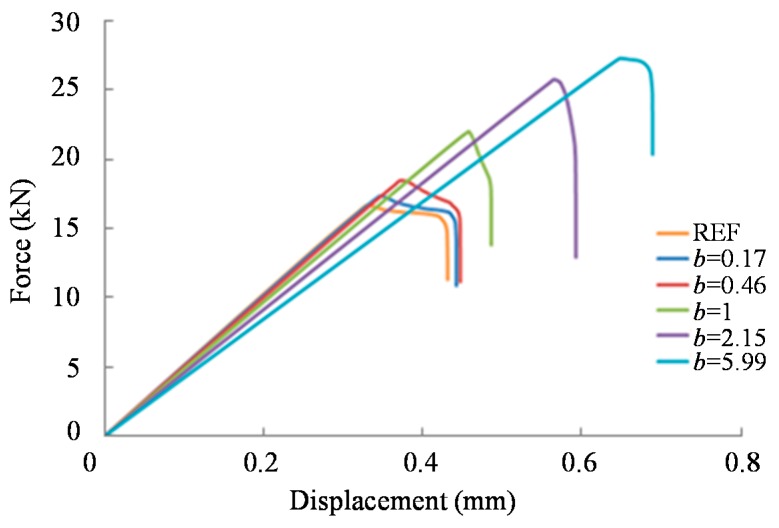
Comparison of the load-displacement response for DLJs with the stiffness in the overlap varying from 20 to 200 GPa (*E_Smin_*/*E_Smax_* equal to 10%; *E_1GFRP_* = *E_Smin_*) and following the parabolic stiffness distribution for different values of the b shape parameter.

Figure 10 shows the load versus displacement results for DLJs with the stiffness in the overlap varying from 20 to 200 GPa (i.e., *E*_Smin_/*E*_Smax_ equal to 10%; *E_1GFRP_* = *E*_Smin_) and following the parabolic stiffness distribution for different values of the *b* shape parameter. Both the reference and bio-inspired joints show a linear response until the maximum load is obtained. As the *b* shape parameter increases, the joints fail at a higher load (see Figure 8 and Figure 10), but become less stiff and failure occurs in a more catastrophic way (Figure 10). According to the simulations, failure initiated at the left edge of the overlap length of the DLJs, in the cohesive zone elements region, just after the peak load was obtained. The damage progression in the cohesive elements was evaluated using the stiffness degradation in shear (SDEG) output variable.

## 4. Conclusions

An overview of natural joint systems has been presented. This literature survey identified a range of different methods that nature uses to create joints between materials. The different natural joining methods were summarised in a taxonomy chart. When joining dissimilar materials, a common solution found in nature is to use a transitional zone of stiffness in the insertion site of the joint, which offers less material mismatch. This biomimetic-inspired solution offers the prospect of reducing stress concentrations and can lead to new engineering joining designs.

A numerical investigation of bio-inspired joints with a transitional zone of stiffness between GFRP and steel adherends has been undertaken to show the potential of such joints in engineering. An optimisation procedure was carried out to identify the material stiffness variation within the steel overlap region which gives the joint with the highest strength. According to the results, the follow conclusions can be drawn:

- An increase in the joint strength was observed as the reduced stiffness of the steel adherend approached the stiffness of the GFRP adherend.
- Compared to reference joints (with no transitional zone of stiffness between the adherends), bio-inspired joints showed a 118% increase of joint strength for the best case.
- The strength increase depends significantly on the form of stiffness variation within the overlap region.
- The potential for increased strength is greater in the double lap and hybrid joints, compared to the single lap joint.

## Figures and Tables

**Table 1:** Material properties of the GFRP adherends.

*E*_1_ (GPa)	*E*_2_ (GPa)	*E*_3_ (GPa)	*ν*_12_	*ν*_13_	*ν*_23_	*G*_12_ (GPa)	*G*_13_ (GPa)	*G*_23_ (GPa)
20	5	5	0.3	0.28	0.28	2	1.5	1.5

**Table 2:** Properties of the adhesive Araldite AV138.

Property	Value
Young’s modulus, *E* (GPa)	15.9
Shear modulus, *G* (GPa)	6.0
Poisson’s ratio, *ν*	0.35
Tensile ultimate strength, *σ_f_* (MPa)	46
Shear ultimate strength, *τ_f_* (MPa)	63
Mode I strain energy release, *G*_IC_ (J/m^2^)	180
Mode II strain energy release, *G*_IIC_ (J/m^2^)	380

## Supplementary Materials

Additional data associated with the analyses are available online at Cambridge University’s repository: http://dx.doi.org/10.17863/CAM.564.

## Abbreviations

The following abbreviations are used in this manuscript:

3D	three dimensional;
CFRP	Carbon fibre reinforced plastic;
CZM	Cohesive zone model;
DLJ	Double lap joint;
CFRP	Carbon fibre reinforced plastic;
GFRP	Glass fibre reinforced plastic;
OL	Overlap length;
REF	Reference;
SLJ	Single lap joint
